# Genotypic and Phenotypic Properties of Cattle-Associated *Campylobacter* and Their Implications to Public Health in the USA

**DOI:** 10.1371/journal.pone.0025778

**Published:** 2011-10-19

**Authors:** Yasser M. Sanad, Issmat I. Kassem, Melanie Abley, Wondwossen Gebreyes, Jeffrey T. LeJeune, Gireesh Rajashekara

**Affiliations:** 1 Food Animal Health Research Program, Ohio Agricultural Research and Development Center, The Ohio State University, Wooster, Ohio, United States of America; 2 Department of Veterinary Preventive Medicine, College of Veterinary Medicine, The Ohio State University, Columbus, Ohio, United States of America; Charité-University Medicine Berlin, Germany

## Abstract

Since cattle are a major source of food and the cattle industry engages people from farms to processing plants and meat markets, it is conceivable that beef-products contaminated with *Campylobacter* spp. would pose a significant public health concern. To better understand the epidemiology of cattle-associated *Campylobacter* spp. in the USA, we characterized the prevalence, genotypic and phenotypic properties of these pathogens. *Campylobacter* were detected in 181 (19.2%) out of 944 fecal samples. Specifically, 71 *C. jejuni*, 132 *C. coli*, and 10 other *Campylobacter* spp. were identified. The prevalence of *Campylobacter* varied regionally and was significantly (*P*<0.05) higher in fecal samples collected from the South (32.8%) as compared to those from the North (14.8%), Midwest (15.83%), and East (12%). Pulsed Field Gel Electrophoresis (PFGE) analysis showed that *C. jejuni* and *C. coli* isolates were genotypically diverse and certain genotypes were shared across two or more of the geographic locations. In addition, 13 new *C. jejuni* and two *C. coli* sequence types (STs) were detected by Multi Locus Sequence Typing (MLST). *C. jejuni* associated with clinically human health important sequence type, ST-61 which was not previously reported in the USA, was identified in the present study. Most frequently observed clonal complexes (CC) were CC ST-21, CC ST-42, and CC ST-61, which are also common in humans. Further, the cattle associated *C. jejuni* strains showed varying invasion and intracellular survival capacity; however, *C. coli* strains showed a lower invasion and intracellular survival potential compared to *C. jejuni* strains. Furthermore, many cattle associated *Campylobacter* isolates showed resistance to several antimicrobials including ciprofloxacin, erythromycin, and gentamicin. Taken together, our results highlight the importance of cattle as a potential reservoir for clinically important *Campylobacter*.

## Introduction

Campylobacteriosis is estimated to affect over one million individuals in the United States annually, with symptoms ranging from mild diarrhea to more serious neuropathies [1]. Of major concern are *C. jejuni*, a species responsible for the majority of human campylobacteriosis, and *C. coli*, which has been exhibiting increased resistance to antimicrobials [2], [3]. Both *C. jejuni* and *C. coli* can readily and asymptomatically colonize major food-animals such as poultry and cattle, subsequently contaminating foods of animal origin including meats and dairy products [4]. Therefore, both species highlight the potential public health impact of *Campylobacter* contamination of food animals.

Although it is known that chickens constitute a major reservoir for *Campylobacter* spp., the occurrence of these pathogens in other food animals such as cattle and its potential impact on human health remain largely uncharacterized. The latter can be partially attributed to the sporadic nature of *Campylobacter* infections and difficulties in isolating these fastidious pathogens. However, since cattle are a major source of food and the multi-faceted cattle industry engages people from farms to processing plants and meat markets, it is conceivable that both live cattle and contaminated cattle products could contribute significantly to *Campylobacter* infections in humans. Furthermore, cattle-associated *Campylobacter* also pose an additional indirect public health risk [5], [6]. For example, contamination of surface and ground water may occur with waste run-off from cattle farming and processing operations. Thus, it is important to further investigate the epidemiology of *Campylobacter* in the cattle population in order to assess associated risks to public health.

Recent studies have shown that the contributions of non-poultry associated *Campylobacter* to human infections were considerable and warrant investigation [7], [8]. For example, evidence collected using Multilocus Sequence Typing (MLST) showed that cases of human *Campylobacter* infections in Finland could be attributed equally to cattle and poultry [9]. Moreover, another study reported that cattle were the source of human infections in 35% of the cases examined in Lancashire, England [8]. This is not surprising since up to 80% of cattle herds and 40–60% of the individual animals shed *Campylobacter* [10], [11], [12]. Consequently, the role of cattle as reservoirs for these pathogens might be important for understanding the epidemiology of *Campylobacter* infections. However, particularly in the US, the prevalence of *Campylobacter* in cattle, their characteristics and relationship to isolates from humans have not been extensively described in the peer reviewed literature.

Host cell invasion and intracellular survival and resistance to antibiotics are important characteristics that affect *Campylobacter* infections in humans. However, the potential role of these properties in facilitating human infections with *Campylobacter* isolated from cattle is not clear. Although epidemiological studies deploy typing analysis to glean information about the relationships between cattle-associated *Campylobacter* and clinically-important human isolates, the capability of cattle-associated *Campylobacter* to invade and persist in the human host might require further assessment, especially when previously unknown sequence types are identified. This also applies to antimicrobial resistance properties, which have been posing a serious concern in *Campylobacter* collected from animal hosts along with the possibility of the transmission of these isolates to humans through the food chain. Therefore, epidemiological studies concerning cattle-associated *Campylobacter* would gain from attempts to amend molecular typing analysis with *in vitro* invasion studies using human intestinal cell lines and phenotypic assays for determining antibiotic resistance.

Since molecular typing data of cattle-associated *Campylobacter* in the United States are limited and little is known about their impact on human health, it is important to adopt a multiphasic approach to characterize cattle-associated *Campylobacter* by using a combination of molecular typing and *in vitro* assays. Therefore, in this study, we determined the genotypes of *C. jejuni* and *C. coli* isolated from cattle slaughtered for meat purpose in different geographical locations in the U.S. and investigated their antimicrobial susceptibility profiles as well as virulence associated phenotypes such as their potential for invasion and persistence in human intestinal epithelial cells.

## Materials and Methods

### Distribution of sampling sites and collection and processing of fecal samples from beef cattle

A total of 944 fresh fecal samples (10 g each) were collected during the summer and early fall of 2008 from cattle presented to slaughter which included both feed-lot (n = 482) as well as mature cows and bulls (n = 462) culled from milking and breeding herds (Table 1). The samples were collected from colon on the conveyer belt during the normal harvest process from slaughtered cattle. To account for potential spatial heterogeneity between different beef processing plants, sampling efforts included seven plants that were distributed across four major geographical locations [North (N), East (E), Midwest (M), and South (S)] in the U.S. The time interval of sample collection per plant spanned two consecutive days and was divided into four periods during which at least four different lots of cattle were sampled in order to capture the genetic diversity of cattle-associated *Campylobacter*. To further limit sampling bias, the number of samples collected per plant was calculated in terms of the total number of animals expected for slaughter on each day, and a statistically calculated sample size to detect at least a single sequence type of *Campylobacter* should it be present in each group of animals at a prevalence of greater than 2.5% with a confidence of 95%. Samples were stored on ice immediately after collection and shipped overnight to the laboratory for *Campylobacter* isolation.

**Table 1:** *Campylobacter* species isolated from cattle fecal samples that were collected from four major geographical locations in the USA.

Regions	Number of samples	*Campylobacter* prevalence/region (%)	Number of *C. jejuni*	Number of *C. coli*
North	210	31/210 (14.8%)	13	16
Midwest	240	38/240 (15.83%)	36	12
South	244	80/244 (32.8%)	8	83
East	250	32/250 (12%)	14	21
Total	944	181/944 (19.2%)*	71	132

*A total of 213 *Campylobacter* isolates occurred in 181 fecal samples including 10 *Campylobacter* spp. other than *C. jejuni* and *C. coli*.

### Isolation and identification of *Campylobacter* species from fecal samples

To isolate *Campylobacter* spp., 1 g of each fecal sample was enriched in Preston broth for 48 h at 42°C under microaerobic conditions (5% O_2_, 10% CO_2_, and 85% N_2_) [13]. From the enrichments, an inoculum (100 µl) was spread onto modified Cefoperazone Charcoal Deoxycholate Agar (mCCDA) plates, which were then incubated for an additional 48 h at 42°C under microaerobic conditions [14]. Three to five colonies suspected as *Campylobacter* were selected from each plate and sub-cultured onto Muller-Hinton agar plates, after which DNA was extracted from each isolate using the Genomic DNA Purification Kit (Epicenter, Madison, WI) as described by the manufacturer. The DNA was then quantified using the NanoDrop 1000 Spectrophotometer V3.7.1 (Fisher Scientific, Pittsburgh, PA) and subjected to species-specific PCR analysis to confirm the identity of the isolates as described elsewhere [15]. PCR analysis targeted a 16S rRNA gene fragment (F) 5′-ATCTAATGGCTTAACCATTAAAC-3′ and (R) 5′-GGACGGTAACTAGTTTAGTATT-3′, *mapA*, (F) 5′-CTATTTTATTTTTGAGTGCTTGTG-3′ and (R) 5′-GCTTTATTTGCCATTTGTTTTATTA-3′ and *ceu*E, (F) 5′ATTTGAAAATTGCTCCAACTATG-3′ and (R) 5′-TGATTTTATTATTTGTAGCAGCG-3′ which indicated the specific detection of *Campylobacter* spp., *C. jejuni*, and *C. coli*, respectively [16], [17]. All PCR products were resolved on a 1.5% agarose gel containing 0.5 µg/ml of ethidium bromide. The size of the PCR products was determined using a 1 Kb DNA ladder and detection was confirmed by comparison to PCR products generated from *C. jejuni* 81–176 (wild-type strain) and *C. coli* (ATCC 33559), which were used as positive controls in all PCR analysis. Negative controls (reactions with no DNA templates) were included in all PCR analyses to ensure specific product amplification.

### Pulsed Field Gel Electrophoresis (PFGE) of *Campylobacter* isolates

To determine their genotypic relatedness, the *Campylobacter* isolated from fecal samples were analyzed using PFGE analysis as described in Ribot et al. [18]. Briefly, *C. jejuni* and *C. coli* isolates were harvested from MH agar plates and suspended to an OD_610_ of 1.4 in 1X PBS. To prepare agarose plugs, OD adjusted suspensions were gently mixed with 1% SeaKem Gold agarose (SKG, Fisher scientific, Pittsburgh, PA) that was pre-melted in TE (10 mM Tris, 1 mM EDTA, pH 8.0). The plugs were then incubated with shaking (200 rpm) in lysis buffer [50 mM Tris, 50 mM EDTA (pH 8.0), 1% sarcosine, 0.1 mg ml^−1^ of proteinase K] for 1 h at 55°C. After lysis the plugs were washed four times and suspended in 5 ml of fresh TE. The plugs were then sliced and digested overnight with *Sma*I at room temperature. The digested slices were loaded onto a 1% SKG agarose gel and DNA fragments were separated by electrophoresis for 20 h using the CHEF Mapper system (Bio-Rad, Hercules, CA) followed by post staining with ethidium bromide. The resulting PFGE patterns were documented and analyzed using the BioNumerics 5.1 software (Applied Maths Inc, Austin, TX). Similarity and clustering analysis of the PFGE patterns were performed using the Dice Coefficient and the unweighted pair-group method with arithmetic averages (UPGMA) with optimization of 1% and position tolerance of 1.5% [7], respectively. The PFGE analysis was also performed on *C. jejuni* 81–176 and *C. coli* (ATCC 33559), which were used as controls for facilitating gel to gel comparison. Furthermore, Lambda Ladder PFG Marker (50–1,000 kb, New England BioLabs, Ipswich, MA) was used as a molecular marker. A cut-off similarity value of 75%, was used to determine the sub-clusters of macro restriction profiles (MRPs).

### Determining the antimicrobial-resistance properties of the *Campylobacter* isolates

Minimal inhibitory concentrations (MIC) for *C. jejuni* and *C. coli* isolates were determined using commercially available 96-well plates containing antimicrobials (Sensititre Campy plates, TREK Diagnostic Systems Inc., Cleveland, OH, USA). These Sensititre Campy plates were used as described by the manufacturer and include *Campylobacter*-relevant antimicrobials such as azithromycin (AZI) (MIC for a resistant *Campylobacter* isolate: ≥8 µg ml^−1^); ciprofloxacin (CIP) (MIC: ≥4 µg ml^−1^); clindamycin (CLI) (MIC: ≥8 µg ml^−1^); erythromycin (ERY) (MIC: ≥32 µg ml^−1^); gentamicin (GEN) (MIC: ≥8 µg ml^−1^); nalidixic acid (NAL) (MIC: ≥64 µg ml^−1^); telithromycin (TEL) (MIC: ≥8 µg ml^−1^); florfenicol (FEN) (MIC: ≥8 µg ml^−1^); and tetracycline (TET) (MIC: ≥16 µg ml^−1^). *Campylobacter* isolates were grown to mid-log phase and then suspended in Mueller-Hinton broth to achieve an OD_600_ of 0.05. For each isolate, one hundred microliter of the suspension were transferred to each well in the Sensititre Campy plates, including a control well that did not contain any antibiotics, while *C. jejuni* 81–176 and *C. coli* (ATCC 33559) were used for quality control. The plates were then incubated under microaerobic conditions at 42°C for 24 h after which the minimum inhibitory concentration (MIC) was measured. The MIC for each antibiotic was defined as the absence of bacterial growth in the well with the lowest concentration of the antibiotic and susceptibility was interpreted according to the Clinical and Laboratory Standards Institute (CLSI) [19].

### Multilocus sequence typing (MLST) of *Campylobacter* isolates

To investigate the clonality of the *Campylobacter* isolates and to assess similarity to strains associated with human infections, MLST analysis was performed on a total of 112 isolates (62 *C. jejuni* and 50 *C. coli*). Nine *C. jejuni* isolates were not typed because either they yielded incomplete profiles or last culturability upon storage. However, since the *C. coli* isolates showed limited genetic diversity using MLST, the tested number of isolates (n = 50) was considered adequate to meet the objectives of this study. These isolates were selected to be representative of different PFGE clusters. MLST analysis was conducted as described by Dingle et al. [20]. Briefly, loci from seven housekeeping genes (*aspA*, *glnA*, *gltA*, *glyA*, *pgm*, *tkt*, and *uncA*) were amplified using gene specific primers by PCR and anticipated sizes of the amplicons were confirmed by agarose gel electrophoresis. The *Campylobacter* MLST oligonucleotides [20] were obtained from Integrated DNA Technologies (Coralville, IA). PCR products were purified (QIA quick 96 PCR purification kit, QIAGEN, Valencia, CA), sequenced in both directions as described previously [21], and, forward and reverse sequences were then aligned using ClustalW (www.ebi.ac.uk/clustalw). Allelic profiles were determined by performing BLAST analysis using the single-locus query function, while sequence types (STs) were assigned using the allelic profile query function available in the MLST *Campylobacter* database (http://pubmlst.org/campylobacter/). STs were then traced to their respective clonal complexes using BURST at http://pubmlst.org/.

### Invasion and Intracellular survival potential of the *Campylobacter* isolates in INT407 cells

Representative *C. jejuni* (n = 19) and *C. coli* (n = 12) that belonged to clonal complexes associated with human infections as well unassigned clonal complexes including the newly identified STs were further tested for their virulence associated phenotypes by assessing their ability to invade and survive within human intestinal epithelial cells. The invasion studies were conducted as described in Konkel et al. [22] and Prasad et al. [23]. Briefly, 10^5^ cells ml^−1^ of INT407 (human embryonic intestine, ATCC CCL 6) were seeded into each well of a 24-well tissue culture plates after suspension in Eagle's Minimum Essential Medium (MEM, Fisher scientific, Pittsburgh, PA) supplemented with 10% fetal bovine serum (FBS, Fisher scientific, Pittsburgh, PA). The plates were then incubated at 37°C in a humidified incubator with 5% CO_2_ until semi-confluent mono-layers were obtained. In preparation for infection with *Campylobacter*, the INT407 mono-layers were washed three times and covered in MEM supplemented with 1% FBS. Similarly, *Campylobacter* cultures were washed three times and suspended in MEM supplemented with 1% FBS to obtain 10^7^ bacteria ml^−1^. One ml of bacterial suspension was added to each well containing the INT407 cell monolayer, achieving a 1∶100 multiplicity of infection (MOI) then incubated for 3 h. After 3 h of incubation with bacteria, cells were treated with gentamicin (150 µg/ml) and incubated for additional 2 h. The infected mono-layers were then washed three times with MEM, lysed using 0.1% Triton X-100 (Fisher scientific, Pittsburgh, PA), serially diluted (10-fold) in MEM and 100 µl of each dilution were spread on MH agar plates. The agar plates were then incubated for 48 h at 42°C under microaerobic conditions, after which colony forming units (CFU) were counted to determine the number of *Campylobacter* that invaded the monolayers. Each isolate was tested in duplicate per assay, and the experiment was repeated three times on separate occasions. *C. jejuni* 81–176 (highly invasive) [24], *C. jejuni* NCTC11168 (poorly invasive) [24] and *C. coli* (ATCC 33559) were used as controls in all invasion assays.

To assess intracellular survival [22], *Campylobacter* cultures and the INT407 cells were processed as described above. However, after treatment with gentamicin, MEM containing 3% FBS and bacteriostatic concentration of gentamicin (10 µg ml^−1^) were added to each well to allow strict quantitation of intracellular bacteria. After further incubation at 37°C for 24 h, the monolayers were washed three times, lysed and serially diluted in MEM as described earlier and plated on MH agar to determine CFU of *Campylobacter* surviving within the intestinal cells. In addition, we also cultured the supernatant of gentamicin treated monolayers to ensure the quality of the gentamicin protection assay. The experiment was repeated three times on separate occasions. *C. jejuni* 81–176, *C. jejuni* NCTC11168, and *C. coli* (ATCC 33559) were used as controls in all intracellular survival assays.

### Statistical analysis

A Chi square test was used to evaluate data collected from the *Campylobacter* prevalence analysis. The data derived from cell culture assays were analyzed using one-way ANOVA followed by Tukey's Multiple Comparison Test. A *P* value of <0.05 was considered statistically significant for all experiments. Measurements expressed as mean ± SE (standard error) were averages of at least three replicas.

## Results

### Occurrence and distribution of *Campylobacter* spp. in feces sampled from cattle

The occurrence and distribution of *Campylobacter* in cattle feces was investigated in order to determine the role of cattle as a potential reservoir for these bacteria. *Campylobacter* were detected in 181 (19.2%) out of 944 fecal samples, while a total of 213 isolates were retrieved from the *Campylobacter*-positive fecal samples. The *C. jejuni* and *C. coli* constituted the majority of the isolates and were detected in 71 (7.5%) and 132 (14%) of the samples, respectively (Table 1). There was no significant difference (*P*<0.12) in the number of *C. jejuni* isolated from fecal samples obtained from; either feed lot (6.2%) or the cull cattle (8.9%), while *C. coli* was more frequently isolated from cull cattle (17%) as compared to feed lot cattle (11%) (*P*<0.01). Furthermore, the occurrence of *Campylobacter* in cattle feces varied according to geographic location of the sampling sites. Specifically, *Campylobacter* species were retrieved from 32.8% of the fecal samples collected from the South, which was significantly higher (*P*<0.05) than those from the North (14.8%), Midwest (15.83%), and East (12%) (Table 1).

### *C. jejuni* and *C. coli* strains were genotypically diverse across different geographic regions

**Figure 1:**
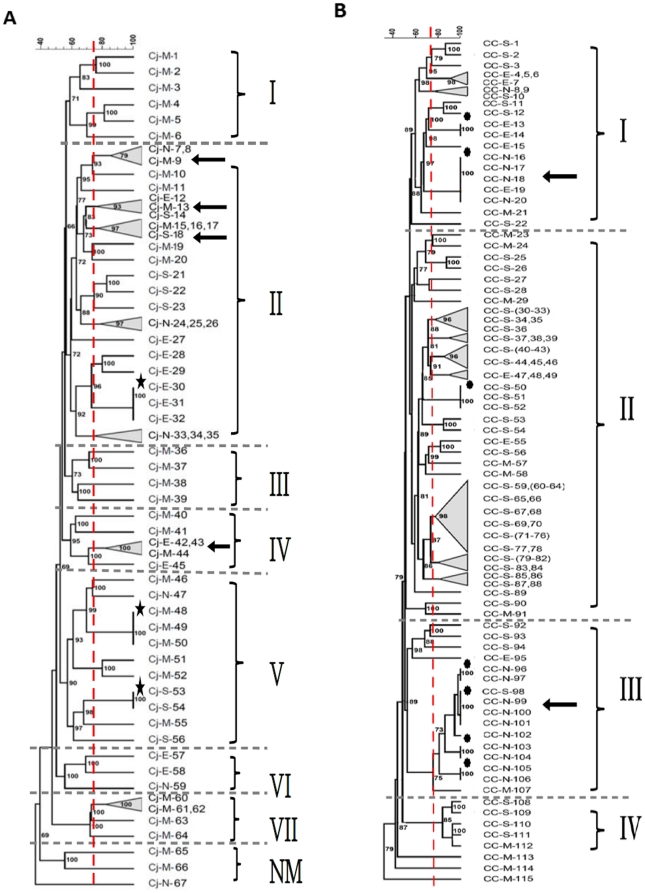
Dendrogram showing the Pulsed-field gel electrophoresis patterns for *Sma*I restricted cattle-associated *C. jejuni* (A) and *C. coli* (B). Similarity analysis was performed using the Dice coefficient, and clustering was performed by the unweighted pair-group method with arithmetic averages UPGMA (optimization, 1% and position tolerance, 1.5%). Clustering cut-off was 75% similarity, which was represented by dashed line. Numbers on bootstraps represent Cophenetic correlations. N = North, S = South, M = Midwest, and E = East. Cj = *C. jejuni*, CC = *C. coli*.

PFGE analysis was performed on *C. jejuni* and *C. coli* isolated from feces in order to determine the genetic diversity of these bacteria and the relationship between the isolates retrieved from different geographic locations. PFGE analysis was successfully performed on 67 out of 71 *C. jejuni*, while 4 isolates could not be typed using the aforementioned method. Regardless, analysis of the PFGE profiles of the *C. jejuni* suggested that the isolates possessed diverse genotypes, especially when comparing profiles of isolates belonging to different geographic locations (Fig. 1A). Furthermore, there were 7 main clusters (Fig. 1A) and by using a cut-off similarity value of 75%, profiles of the *C. jejuni* were classified to15 sub-clusters. With the exception of 4 clusters that included isolates that were collected from 2 to 3 different geographic locations, *C. jejuni* profiles were mostly found to form geographically homogenous groupings. For example, one cluster was composed of three isolates collected from the East, the Midwest, and the South, respectively, while others included 4 isolates, 3 of which were isolated from the Midwest and one from the South (see arrows in Fig. 1A). Overall, it was interesting to observe that profiles of the isolates from the North did not group with those collected from the East and the South.

Similarly, PFGE analysis was successfully performed on 115 out of 132 *C. coli* isolates, while the remaining isolates could not be typed using the aforementioned method (Fig. 1B). Unlike the *C. jejuni*, PFGE profiles of certain *C. coli* isolated from all four geographic locations were found to share a 100% similarity. Specifically, analysis grouped the *C. coli* isolates into 4 main clusters and by using a cut-off similarity value of 75%, 21 sub-clusters were identified, 2 of which included identical profiles (100% similar) for isolates from different geographic locations (See arrows Fig. 1B). For example, a cluster was composed of 5 isolates (4 from the North and 1 from the East) that displayed identical profiles, while another cluster similarly contained identical profiles of 4 isolates (3 from the North and 1 from the South). However, other clusters contained profiles of *C. coli* isolates from different geographic locations that were not identical, yet exhibited high similarity (between 79 to 95%) than that of the cut-off (75%). Similar to PFGE analysis of *C. jejuni*, many *C. coli* isolated from the same geographic location showed profiles that tend to cluster together.

### Antimicrobial susceptibility of *C. jejuni* and *C. coli* isolates

**Figure 2:**
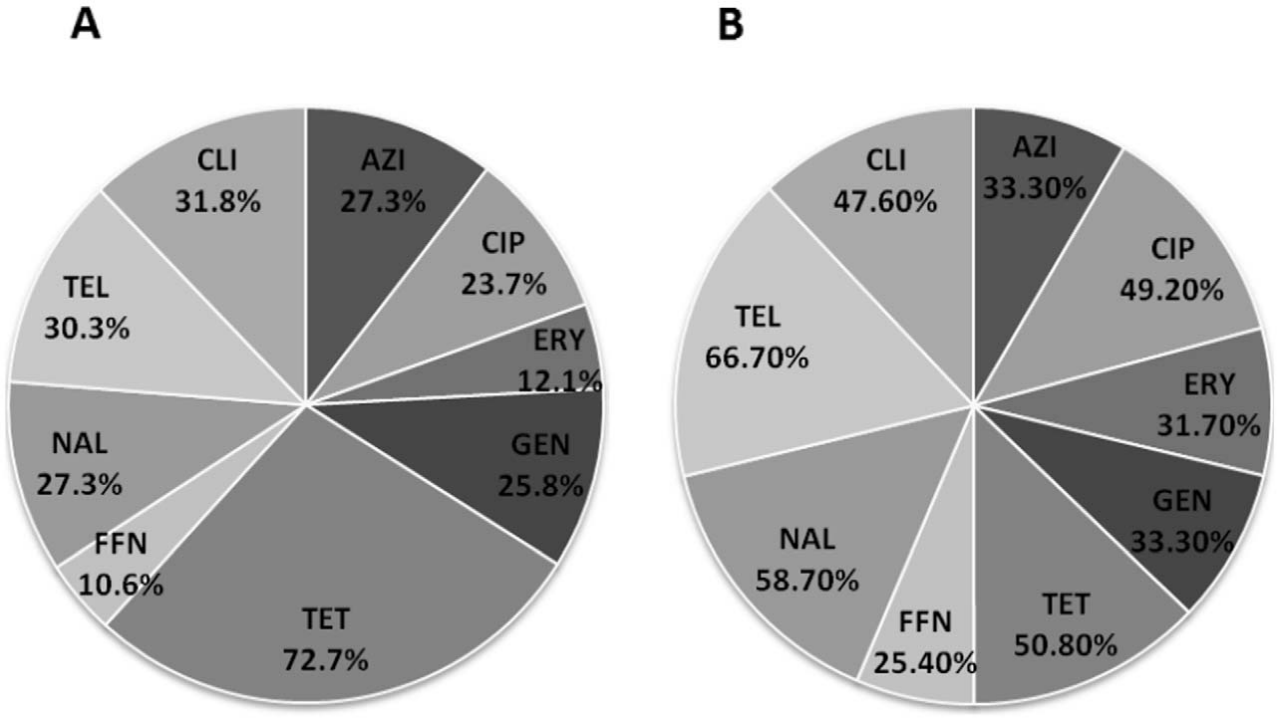
Antimicrobial resistance of *Campylobacter* isolated from cattle. *C. jejuni* (**A**) and *C. coli* (**B**) isolates obtained from four different locations across the US. Percentage of isolates resistant to different antimicrobials used in this study is shown. *C. jejuni* 81–176 and *C. coli* ATCC 33559 strains were used for quality control.

To better assess the potential public health impact of the *Campylobacter* spp. associated with cattle, the isolates were assayed for their potential to resist antibiotics that are of both clinical and veterinary importance. Antimicrobial susceptibility was assessed on 66 (the 5 remaining isolates lost cultivability after storage) *C. jejuni* isolates using commercially available Sensititre Campy plates. The *C. jejuni* isolates were resistant to different antimicrobials including, ciprofloxacin (MIC: 4–64 µg ml^−1^), erythromycin (MIC: 32 µg ml^−1^), tetracycline (MIC: 16–64 µg ml^−1^), and clindamycin (MIC: 8–16 µg ml^−1^) (Fig. 2A). However, resistance to tetracycline was observed for the majority of the tested *C. jejuni* (72.7%), while resistance to nalidixic acid was observed for only 27.3% of the isolates (Fig. 2A). Furthermore, 10 (15.1%) tested *C. jejuni* were resistant only to tetracycline, while 34 (51.5%) isolates were resistant to 3 or more antimicrobials, including ciprofloxacin, tetracycline, nalidixic acid, erythromycin, and clindamycin. Overall, a higher percentage of *C. jejuni* isolates from the South region showed decreased resistance to most of the antimicrobials (See asterisk Table 2). The MRPs and antimicrobial resistance profiles of the *C. jejuni* isolates analyzed by MLST are summarized in Table S1.

**Table 2:** Antimicrobials resistance of *C. jejuni* and *C. coli* isolated from four different locations.

	Number of resistant isolates (%) and range of MICs in µg/ml									
Isolates		AZI	CIP	ERY	GEN	TET	FFN	NAL	TEL	CLI
**CJ**	**N**	5(7.6%)8–32^a^	6(9.1%)4–64	1(1.5%)32	3(4.5%)8–16	9(13.6%)16–64	3(4.5%)8–16	8(12.1%)64	7(10.6%)8	4(6.1%)16
	**S** *	0	1(1.5%)64	0	0	2(3.0%)16–64	0	2(3.0%)64	0	0
	**M**	6(9.1%)8–16	4(6.1%)16–32	4(6.1%)32	8(12.1%)8–16	26(39.4%)16–64	4(6.1%)8–16	4(6.1%)64	9(13.6%)8	11(16.7%)8–16
	**E**	7(10.6%)8–32	4(6.1%)16–32	3(4.5%)32	6(9.1%)8–16	11(16.7%)16–64	0	4(6.1%)64	4(6.1%)8	6(9.1%)8–16
**CC**	**N**	0	2(3.2%)4–16	0	1(1.6%)8	1(1.6%)16	0	6(9.5%)64	4(6.3%)8	2(3.2%)8–16
	**S**	10(15.9%)8–64	24(38.1%)8–64	10(15.9%)32–64	13(20.6%)8–32	20(31.7%)16–64	10(15.9%)8–32	23(36.5%)64	26(41.3%)8	16(25.4%)8–16
	**M**	3(4.8%)64	2(3.2%)8–32	2(3.2%)32–64	4(6.3%)8–16	3(4.8%)16–64	0	2(3.2%)64	5(7.9%)8	3(4.8%)16
	**E**	8(12.7%)16–64	3(4.8%)4–8	8(12.7%)32–64	3(4.8%)8–16	8(12.7%)16–32	6(9.5%)8–64	6(9.5%)64	7(11.1%)8	10(15.9%)8–16

a resistance MIC range of antimicrobials.

*decreased resistance to most of antimicrobials.

Since, *C. coli* isolates belonging to different PFGE clusters were highly similar, we tested representative isolates from each cluster for a total of 63 isolates. Although the range of MIC to disparate antibiotics was at instances different from those observed for *C. jejuni*, the *C. coli* isolates also exhibited resistance to the several of the antimicrobials tested (Fig. 2B). For example, *C. coli* isolates were resistant to ciprofloxacin (MIC: 4–64 µg ml^−1^), erythromycin (MIC: 32–64 µg ml^−1^), tetracycline (MIC: 16–64 µg ml^−1^), and clindamycin (MIC: 16 µg ml^−1^) (Table 3). Furthermore, resistance to telethromycin was observed for the majority of the tested *C. coli* (66.7%), while resistance to flourofenicol was observed for only 25.4% of the isolates (Fig. 2A). Of the 63 tested *C. coli*, 3 (4.8%), 9 (14.3%) and 48 (76.2%) isolates were resistant to only one (e.g. nalidixic acid), two (e.g. ciprofloxacin and nalidixic acid), and three or more antimicrobials, respectively. Furthermore, a higher percentage of *C. coli* isolates from the South showed increased resistance to all the antimicrobials (Table 2).

**Table 3:** Distribution of multilocus sequence types among cattle *C. jejuni* and *C. coli*.

ST*	*C. jejuni* Isolates	Isolates/Region				ST- CC
		N	E	M	S	
590	3	0	1	2	0	**ST-42**
1013	11	2	0	7	2	
459	5	0	0	5	0	
797	7	0	1	5	1	**ST-21**
4924	5	0	0	3	2	
2876	2	0	0	2	0	
4026	2	1	0	1	0	
4930	1	0	1	0	0	
4922	1	0	0	1	0	
4923	1	0	0	1	0	
239	1	0	0	1	0	
61	2	0	2	0	0	**ST-61**
500	1	0	0	1	0	
3091	1	1	0	0	0	**ST-45**
3084	1	0	0	0	1	**ST-48**
4929	2	0	2	0	0	**Unassigned**
4925	1	0	0	1	0	
4926	1	0	0	1	0	
4927	1	1	0	0	0	
4928	1	0	1	0	0	
4931	1	0	0	1	0	
4932	1	0	0	0	1	
922	1	0	1	0	0	
5447	2	0	0	2	0	
5448	1		1			

ST	*C. coli* Isolates	Isolates/Region				ST- CC
		N	E	M	S	
1068	40	8	2	7	23	**ST-828**
902	2	0	0	2	0	
2501	2	1	0	0	1	
1110	2	0	2	0	0	
3866	1	0	0	0	1	
5372	1	0	0	0	1	
4933	1	0	0	0	1	
5446	1	0	0	1	0	

*Complete allelic profiles for six *C.jejuni* isolates could not be ascertained.

### MLST analysis identifies STs belonging to human clonal complexes

To determine the genotypic properties of *Campylobacter* spp. isolated from cattle as well as their relationship to those of human origin, MLST analysis was performed on selected *C. jejuni* and *C. coli* isolates. A total of 112 isolates, 62 *C. jejuni* and 50 *C. coli* that represented the main PFGE clusters and different geographic locations, were selected for MLST analysis. Thirteen new *C. jejuni* STs were identified and designated as STs 4922 to 4932, 5447, and 5448 (Table 3). These new STs were recognized following query with the PubMLST database. Furthermore, three new alleles, *gly*A; 416 (Cj-M-46), *pgm*; 537 (Cj-M-63) and *tkt*; 436 (Cj-M-64) were detected for 3 STs out of 13 new *C. jejuni* STs. Both *pgm* and *tkt* alleles had only a single nucleotide polymorphism, while *gly*A had a two nucleotide polymorphism.

In general *C. jejuni* isolates showed high genetic diversity by MLST analysis (Table 3). A total of 25 different STs were identified. Twenty isolates with 8 STs belonged to Clonal Complex (CC) ST-21, while 3 of these isolates were assigned to new STs. Additional isolates (n = 19) were grouped into 3 STs that belonged to CC ST-42, while five, three, and 11 isolates were identified as ST-459 which belonged to CC ST-42, ST-590, and ST-1013, respectively. Finally, one isolate belonged to CC ST-45, one belonged to CC ST-48, and 9 belonged to undefined CC (Table 3). Interestingly, we also detected in our study three isolates belonging to clonal complex ST-61, and two of them were identified as ST-61, which is clinically important and has not been reported previously in cattle in the USA according to the PubMLST database. The most frequently observed clonal complexes (CC) were CC ST-21, CC ST-42, and CC ST-61, which are also common in humans. Six isolates in our collection had incomplete allelic profiles and could not be assigned to any ST.

In contrast to *C. jejuni* isolates, *C. coli* isolates showed very limited diversity (Table 3). Of the 50 *C. coli* isolates analyzed (Table 3), a total of 8 STs were identified with 1 isolate belonging to new ST-4933 and ST-5446. Interestingly, 40 (80%) isolates were identified as ST-1068. Twenty three of those 40 isolates were isolated from the South as well as 8 from the North, 7 from the Midwest and 2 from the East. Additionally, seven different STs were identified from the remaining 10 strains that were isolated from all four regions. Two of these isolates were identified as ST-902 (from Midwest), while the remaining two isolates (from the North and the South, respectively) were identified as ST-2501. Additional isolates (n = 2) were identified as ST-1110 (East), while three more isolates were identified as ST-3866, ST-5372, and ST-4933, respectively. Additionally, one isolate was identified as a new ST-5446 (Midwest). All 50 *C. coli* isolates belonged to CC ST-828 (Table 3).

### Invasion and intracellular survival potential of cattle associated *C. jejuni and C. coli*

**Figure 3:**
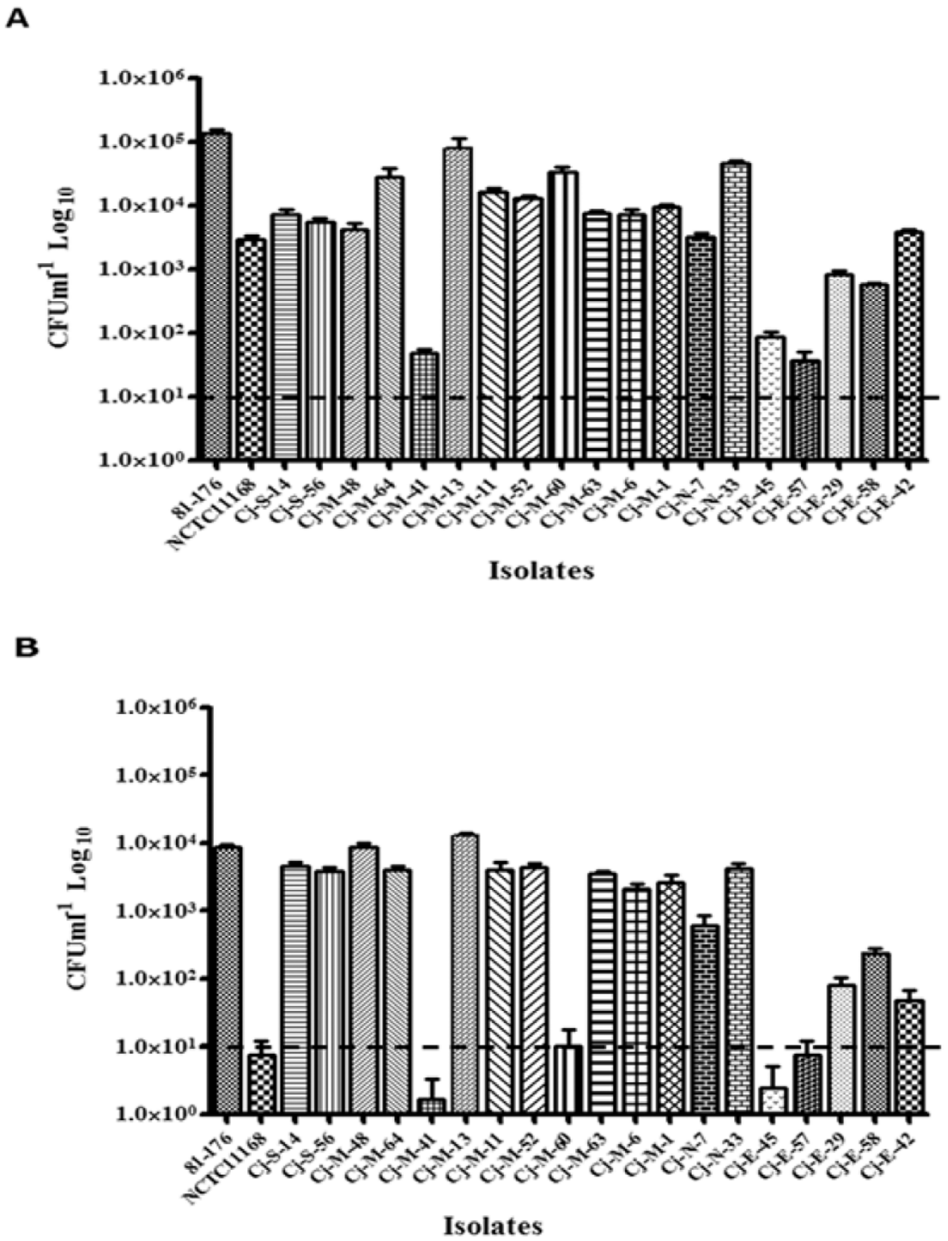
Invasion and intracellular survival of cattle *C. jejuni* isolates in INT407 cells. **A.** CFU ml^−1^ representing the number of the internalized bacteria which could be retrieved after treatment of cells with gentamicin. **B.** Intracellular survival of *C. jejuni* isolates in INT407cells. CFU ml^−1^ representing the numbers of internalized bacteria retrieved after 24 h of incubation following the gentamicin treatment. The INT407 were infected with 1∶100 MOI of *C. jejuni* strains. *C. jejuni* 81–176 and NCTC11168 were used as controls. The detection limit of the assay is represented by the dashed line. Each bar represents the mean ± SE of three independent experiments done in duplicates for each sample (*P*<0.01).

To investigate the virulence-associated potential of the cattle-associated *C. jejuni* and *C. coli*, we tested selected isolates for their ability to invade and survive in human INT407 intestinal epithelial cells. *C. jejuni* and *C. coli* isolates that belonged to all the detected CCs were selected for these assays; in addition isolates that exhibited disparate antibiotic resistance profiles and/or originated from different geographic locations were also included in this analysis. All tested *C. jejuni* isolates (n = 19) invaded INT407 (Fig. 3A); however, the invasion potential varied between isolates, ranging from an average of 2.75×10^1^ to 8.0×10^4^ CFU ml^−1^ (Fig. 3A). The invasion capability of 17 of the 19 isolates tested was significantly lower than that of the highly invasive strain *C. jejuni* 81–176 (*P<0.01*); however, 11 of these isolates invaded the INT407 cells with higher numbers as compared to the poorly invasive *C. jejuni* NCTC11168 (Fig. 3A). Furthermore, two *C. jejuni* isolates Cj-M-13 and Cj-N-33 exhibited high invasion potential that matched that of *C. jejuni* 81–176 (Figure 3A). Interestingly, all tested *C. jejuni* isolates were capable of intracellular survival, albeit the potential varied between isolates, ranging from an average of 8.0×10^1^ to 1.3×10^4^ CFU ml^−1^(Fig. 3B). Additionally, the same aforementioned two isolates showed a significantly higher (*P*<0.01) potential for intracellular survival as compared to that of *C. jejuni* 81–176.

**Figure 4:**
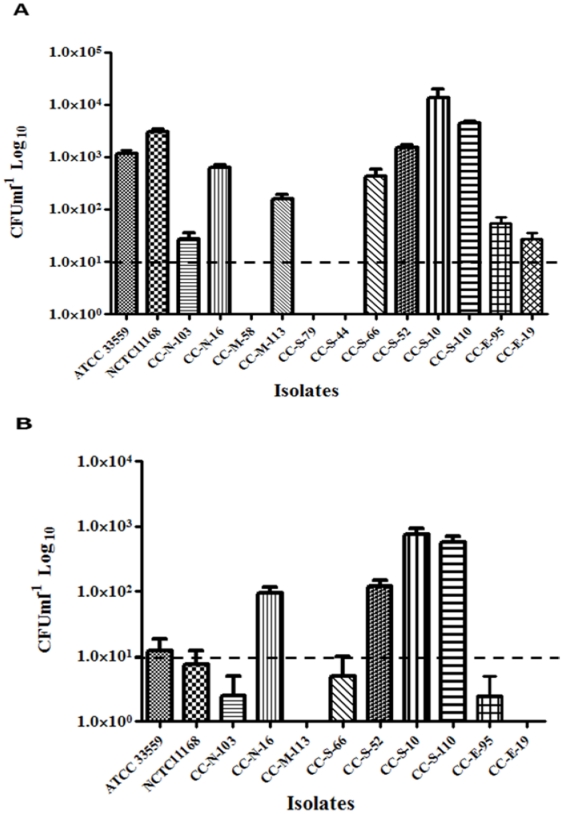
Invasion and intracellular survival of cattle *C. coli* isolates in INT407 cells. **A.** CFU ml^−1^ representing the number of the internalized bacteria which could be retrieved after treatment of cells with gentamicin. **B.** Intracellular survival of *C. coli* isolates in INT407cells. CFU ml^−1^ representing the numbers of internalized bacteria retrieved after 24 h of incubation following the gentamicin treatment. The INT407 cells were infected with 1∶100 MOI of *C. coli* strains. *C. coli* (ATCC 33559) and *C. jejuni* NCTC11168 were used as controls. The detection limit of the assay is represented by the dashed line. Each bar represents the mean ± SE of three independent experiments done in duplicates for each sample (*P*<0.01).

On the other hand, only nine *C. coli* isolates could invade the INT407 cells with average numbers ranging between 2.75×10^1^ and 1.34×10^4^ CFU ml^−1^ (Fig. 4A). There was a significant difference in the invasion capabilities between the *C.coli* isolates (*P<0.05*). Furthermore, only seven isolates were capable of intracellular survival (Fig. 4B). Three isolates that did not invade were not tested for intracellular survival. In general *C. coli* isolates were less invasive and displayed reduced intracellular survival compared to *C. jejuni*.

Our data also show that invasive *Campylobacter* possessed a variable antimicrobial phenotype that ranged from complete susceptibility to resistance to 7 different antimicrobials (Table 4). However, it was notable that *C. jejuni* isolates with high invasion potential (Cj-M-13 and Cj-N-33) were also resistant to multiple antimicrobials (see asterisks Table 4; Fig. 3A).

**Table 4:** The properties of *C. jejuni* and *C. coli* isolatesthat were tested for invasion and intracellular survival in INT407 cell line.

*C.jejuni* Isolates	Invasion^a^	Survival	Antimicrobial Resistance Profile	ST	CC ST	MRP cluster
Cj-M-6	Moderate	High	Susceptible	797	CC ST21	II
Cj-M-11	Moderate	High	GEN, TET, NAL	4922	CC ST21	II
Cj-M-52	Moderate	High	CIP, TET, TEL, CLI	4924	CC ST21	V
Cj-M-13*	High	High	AZI, CIP, ERY, GEN, FFN, TEL, CLI	2876	CC ST21	II
Cj-N-33*	High	High	AZI, CIP, GEN, TET, NAL, TEL, CLI	4026	CC ST21	II
Cj-M-48	Moderate	High	ERY, CLI	4923	CC ST21	V
Cj-M-41	Poor	Poor	TET, FFN, NAL	239	CC ST21	IV
Cj-E-29	Poor	Moderate	CIP, TET, TEL	4930	CC ST21	II
Cj-M-60	Moderate	Poor	TET	459	CC ST42	VII
Cj-S-14	Moderate	High	Susceptible	4924	CC ST21	II
Cj-E-58	Poor	Moderate	AZI, GEN, TET, NAL, TEL	4929	UA	VI
Cj-M-63	Moderate-High	High	TET	4925	UA	VII
Cj-M-1	Moderate	High	TET, TEL, CLI	4931	UA	I
Cj-M-64	Moderate-High	High	AZI, CIP, ERY, GEN, TET, CLI	4926	UA	VII
Cj-N-7	Poor	Moderate-High	AZI, ERY, FFN, TEL, CLI	4927	UA	II
Cj-E-57	Poor	Poor	CIP, ERY, TET	922	UA	VI
Cj-E-42	Poor	Moderate	CIP, GEN, TET, NAL, CLI	4928	UA	IV
Cj-S-56	Moderate	High	TET	4932	UA	V
Cj-E-45	Moderate	Poor	AZI, ERY, GEN, CLI	61	CC ST61	IV

*C.coli* Isolates	Invasion	Survival	Antimicrobial Resistance Profile	ST	CC ST	MRP cluster
CC-N-103	Poor	Poor	NAL	1068	CC ST828	III
CC-N-16	Poor	Moderate	NAL	1068	CC ST828	I
CC-M-58	Non	N/A	AZI, GEN, TEL, CLI	902	CC ST828	II
CC-M-113	Poor	Non	CIP, TET, NAL, TEL	1068	CC ST828	IV
CC-S-79	Non	N/A	CIP, TET, NAL, TEL	1068	CC ST828	II
CC-S-44	Non	N/A	CIP, TET, NAL, TEL	1068	CC ST828	II
CC-S-66	Poor	Poor	CIP, TET, TEL	1068	CC ST828	II
CC-S-52	Poor	Moderate	AZI, ERY, GEN, FFN, TEL, CLI	2501	CC ST828	II
CC-S-10	Moderate	Moderate- high	AZI, ERY, GEN, TET, TEL, CLI	5372	CC ST828	I
CC-S-110	Poor	Moderate- high	CIP, TET, NAL	4933	CC ST828	IV
CC-E-95	Poor	Poor	AZI, ERY, GEN, TET, FFN, NAL, TEL, CLI	1110	CC ST828	III
CC-E-19	Poor	Non	AZI, ERY, GEN, CLI	1068	CC ST828	I

a Classification index: High =  higher or comparable to *C. jejuni* 81-176. Moderate-High: Significantly lower than *C. jejuni* 81-176 but significantly higher than moderate. Moderate: Significantly higher than *C. jejuni* NCTC11168. Poor: significantly lower or comparable to *C. jejuni* NCTC11168.

## Discussion

We investigated the occurrence of two important *Campylobacter* spp. (*C. jejuni* and *C. coli*) in cattle that are slaughtered for meat production across 4 major geographic locations in the USA. This is in contrast to previous studies of cattle-associated *Campylobacter* in the USA, which have been mostly geographically confined and/or limited to investigating the prevalence of certain species as well as other phenotypic characteristics such as antibiotic resistance [12], [25], [26]. Therefore, our approach was unique as it included analysis of genotypic diversity and antimicrobial resistance properties as well as attempts to understand the potential role of cattle-associated *Campylobacter* in virulence related phenotypes that might impact human health. Significantly, our findings highlight the importance of cattle as a reservoir for genotypically diverse, antimicrobial resistant, and potentially virulent *Campylobacter* in the USA.

Although the association of *Campylobacter* spp. with cattle in the USA has been investigated previously, available literature reported differing prevalence numbers for these pathogens. For example, Hoar et al [26] isolated *Campylobacter* from 5% of the fecal samples collected from cattle, while Sato et al [27] and Gharst et al [28] showed that these bacteria occurred in 27.9% and 23.4% of their samples, respectively. These differences in the prevalence of cattle-associated *Campylobacter* can be attributed to several factors, including methods for isolation, sample size and type (e.g. dairy versus feedlot), seasonal variations, and geographical location [2]. However, the latter factor, geographical location, was a prominent difference in the aforementioned prevalence studies, which were conducted in California, Wisconsin and Southeastern region of the USA, respectively [26], [27], [28]. Since the prevalence of cattle-associated *Campylobacter* in the USA seemed to vary according to the geographic location, it was necessary in this study to investigate this assumption as well as the overall occurrence of these bacteria in cattle. Our results showed that the occurrence of *Campylobacter* in Southern USA (prevalence of 32.8%) was significantly (*P*<0.05) higher than those observed in Northern (14.8%), Midwestern (15.83%), and Eastern (12%) locations. The precise reasons that contribute to the observed differences in geographic distribution of the *Campylobacter* are not clear. Nevertheless, previous studies have suggested that environmental conditions such as temperature, humidity, and sunlight might contribute to increases in human infections with *Campylobacter* [29]. Subsequently, the climate might be a driver of the relatively higher prevalence of cattle-associated *Campylobacter* in the Southern USA, which is generally warmer as compared to the other locations. Interestingly, Stanley et al. [30] suggested that indirect-temperature dependent factors such as migratory animals might impact the occurrence of *Campylobacter* in dairy cattle. Additionally, *C. coli* have been previously reported to predominantly occur in samples collected from broiler flocks during warmer months [15]. Strikingly higher prevalence of *C. coli* (83 isolates) in our samples from the South (Table 1) further support a role for climate in affecting the geographic prevalence of *Campylobacter*. Regardless, the overall prevalence of cattle-associated *Campylobacter* in this study was 19.2%, falling between the percentages (0.8 to 46.7%) reported for cattle from other countries [31]. However, it is important to note that samples in our study were collected only over a two-day period from a limited number of locations in each region. Furthermore, to emphasize a public health relevance of cattle as potential reservoir for *Campylobacter* (discussed below), our sampling efforts focused on the seasons that normally witness an increase in *Campylobacter* incidences [8]. As such our prevalence data don't account for possible seasonal and temporal variations and, from this study alone, the extent to which differences between geographic locations contributed to *Campylobacter* prevalence in cattle cannot be conclusively ascertained. Nevertheless, our prevalence data provide evidence that like in other countries cattle in the USA might constitute a considerable reservoir for these pathogens, promoting their potential persistence in cattle-derived products and environment, which might pose a risk for human consumers and cattle-handlers.

The genus *Campylobacter* contains species that are known for their adaptation capabilities, readily acquiring genetic material necessary for survival and persistence [32]. High genotypic diversity has been reported in *Campylobacter* isolated from humans, chickens, sheep, pigs, and cattle [33], [34], [35], [36]. This is important since the genotypic diversity of these bacteria might serve as an indicator for the value of a reservoir in providing a suitable niche for the persistence of these microorganisms and their evolution [36]. As elegantly demonstrated by Wilson et al. [36], the broiler gastrointestinal tract constitutes such a niche, allowing multiplication of *C. jejuni* and enhancing its genetic diversity. Subsequently, it was important to investigate the genetic diversity of *Campylobacter* in cattle in order to assess the potential attributes of this reservoir. Our PFGE analysis of the cattle-associated *Campylobacter* revealed that all *C. jejuni* and *C. coli* isolates were generally grouped into 7 and 4 major clusters, respectively (Fig. 1). Remarkably, some *C. jejuni* isolates possessed genotypes that were 100% similar on 3 different occasions (see stars in Fig. 1A). Similarly, several *C. coli* exhibited 100% similar genotypes and were encountered on seven occasions (see asterisks in Fig. 1B). Taken together, these observations confirm a high genetic diversity within the cattle-associated *C. jejuni* and *C. coli* isolated in the USA, which also corroborates the findings of studies conducted on cattle in other countries, including Spain [35], the United Kingdom [37], Denmark [38], Finland [9], and Turkey [39]. Additionally, our PFGE analysis revealed clustering of some *C. jejuni* isolates (more than 75% similarity) that originated from different geographic locations. Likewise, some clusters included *C. coli* isolates with identical genotypes originating from different geographic locations (see arrows Fig. 1). This indicated that isolates with identical genotypes can occur in different herds, which highlights the possibility for certain strains to be transmitted among disparate herds/individuals. However, the latter requires further investigation to elucidate potential routes and mechanisms of transmission. Our PFGE analysis shows that cattle can be considered a potential reservoir for genetically diverse *Campylobacter* that is worth investigating in order to determine its relevance to public health in the USA.

An outstanding feature in livestock associated *Campylobacter* is its ability to resist antibiotics (reviewed in Luangtongkum et al. [40], an increasingly important emerging threat to public health [41]. Furthermore, previous studies have demonstrated that cattle-associated *Campylobacter* in the USA can potentially resist front-line therapeutic drugs for treating human infections. For example, 31.8% and 44% of *C. coli* collected from calves and feedlot cattle were resistant to erythromycin and ciprofloxacin, respectively [31]. Additionally, 47.7% and 49.1% of *C. jejuni* isolated from dairy and feedlot cattle, respectively, were resistant to tetracycline [25], [42]. In agreement with these studies, our antibiotic resistance analysis showed that a high percentage of the cattle-associated strains were resistant to erythromycin, ciprofloxacin, tetracycline and others (Fig 2A and Table 2). Remarkably, 23.7% and 12.1% of the cattle-associated *C. jejuni* that were analyzed in our study exhibited resistance to ciprofloxacin and erythromycin, respectively (Fig. 2A). This was an important and marked difference when compared to previous studies in the US that reported a relatively lower frequency of resistance to ciprofloxacin (1.8% to 5%) and erythromycin (0.4% to 2.9%) in *C. jejuni* isolated from different cattle operations [25], [31], [42]. Our data further support previous findings and predictions that livestock-associated *Campylobacter* are becoming increasingly resistant to important antibiotics [41], [43]. These observations highlight the need for rigorous surveillance of antibiotics used in cattle operations in order to facilitate interventions and curb further emergence of antibiotic resistant *Campylobacter*.

Understanding the contributions of cattle to human infections with *Campylobacter* relies heavily on: i) assessing the relationship between the cattle-associated *Campylobacter* and those isolated from- or previously implicated in human infections and ii) directly examining the potential of these isolates to invade and persist in the human intestine. However, these two criteria were rarely investigated together in previous research that focused on cattle-associated *Campylobacter* and are, to our knowledge, absent in similar studies conducted in the USA. To meet the aforementioned criteria, we typed a pool of the cattle-associated *Campylobacter* using MLST and further analyzed a subset of isolates for their properties in an *in vitro* surrogate for the human host (i.e. INT407 intestinal cell line). Since interpretation of the MLST typing depends on pre-existing online data bases (PubMLST) to assign sequence types (STs) to the isolates, it is possible to compare the cattle-associated *Campylobacter* from this study to others from different sources (e.g. humans) and locations, including other countries. Subsequently, our MLST analysis confirmed that cattle-associated *C. jejuni* (n = 62) were diverse and belonged to disparate STs (n = 25; Table 3), while only 8 STs were assigned to *C. coli* (n = 50) (Table 3). Furthermore, we identified 12 *C. jejuni* that belonged to unassigned clonal complexes (n = 10), which along with newly discovered alleles (n = 3) and STs (n = 13) (Table 3) emphasize the genetic diversity of this bacterium in cattle, possibly indicating that certain *C. jejuni* might be highly associated with this potential source of infection. The latter is supported by reports showing that CC ST-21 and ST-61 were predominant in cattle samples from other countries, including the UK, Canada, New Zealand, and Finland [9], [33], [44], [45]. The detection of CC ST-61 and CC ST-21 (Table 3) in this study was of particular interest, since these complexes constitute two of six CCs that harbor 60% of *C. jejuni* associated with human disease [20], [37]. Although all tested cattle-associated *C. jejuni* were capable of invading the human INT407 cells with variable efficiency, two isolate (Cj-M-13) and (Cj-N-33) that belong to CC ST-21 not only exhibited an invasive potential that was comparable to the hyper-invasive strain *C. jejuni* 81–176 [46] but also showed a higher capability to survive in the intracellular milieu (Fig. 3). Furthermore, these two isolates were resistant to 7 out of 9 tested antimicrobials, including ciprofloxacin, erythromycin, and gentamicin (Table 4). Along the same lines, 11 out of 19 *C. jejuni* with varying invasive capacities were able to either match or outperform the intracellular survival potential of *C. jejuni* 81–176 (Fig. 3). No specific association was observed with either invasion or intracellular survival of the tested *C. jejuni* isolates and their MRPs, antimicrobial resistance properties or their geographical distributions. Since many of the tested *C. jejuni* (13 out of 19) were moderate to highly invasive (Table 4) and given this bacterium's aforementioned capability for natural transformation, the occurrence of invasive strains in a reservoir that is witnessing a rise in antibiotic-resistant *Campylobacter* constitutes a cause for caution and increased surveillance. Since invasion and intracellular survival of pathogens facilitates evading the immune system of the host and allows for persistent and recurrent infection [47], it can be argued that the cattle-associated *C. jejuni* would pose a plausible risk to human health.

Furthermore, MLST data and invasion studies also showed relatively lower clinical relevance of selected cattle-associated *C. coli* isolates, which all belonged to CC ST-828, a complex that include strains which are mainly isolated from agricultural and environmental sources and some from clinical cases [48]. Although the invasion and intracellular survival capacities of *C. coli* were overall lower than those of *C. jejuni*, one cattle associated *C. coli* (CC-S-10) invaded the cells with approximately 10^4^ CFU per ml, while also surviving inside the cells in relatively high numbers (Fig. 4). Taken together, our data confirm the observations of Sheppard et al. [48] that ruminants also are among the most likely sources of human infections with *C. coli*.

We conclude that cattle in the USA constitute a suitable niche for the persistence of *Campylobacter* and a potentially important source for human infections with these pathogens. The potential of cattle-associated *Campylobacter* to resist antibiotics, invade and persist in human cells, and their genetic classification into clinically important clonal complexes warrant highlighting the contribution of cattle to the epidemiology of these pathogens in the USA.

## Supporting Information

Table S1 **Antimicrobial resistance profiles, Sequence Types, and MRP clusters of** ***C.jejuni*** **isolates.**
(DOC)
