# Supplementary material for: Genotypic and Phenotypic Properties of Cattle-Associated Campylobacter and Their Implications to Public Health in the USA
Source: PLoS One. 2011 Oct 19;6(10):e25778. doi: 10.1371/journal.pone.0025778 (PMC3198382; doi:10.1371/journal.pone.0025778)
Supplement: Table S1 — Antimicrobial resistance profiles, Sequence Types, and MRP clusters of C.jejuni isolates. (DOC) [file pone.0025778.s001.doc]

| **Table S1.** Antimicrobial resistance profiles, Sequence Types, and MRP clusters of *C.jejuni* isolates. | | | | |
| --- | --- | --- | --- | --- |
|  | | | | |
| **Isolates^a^** | **Antimicrobial Resistance Profile** | **ST** | **CC ST** | **MRP^b^ cluster** |
| Cj-M-44 | TET | 797 | CC ST21 | IV |
| Cj-M-19 | TET | 797 | CC ST21 | II |
| Cj-M-16 | AZI, GEN, TET, TEL, CLI | 797 | CC ST21 | II |
| Cj-M-36 | AZI, GEN, TET, CLI | 797 | CC ST21 | III |
| Cj-E-28 | AZI, TET | 797 | CC ST21 | III |
| Cj-M-52 | CIP, TET, TEL, CLI | 4924 | CC ST21 | V |
| Cj-M-49 | GEN, TET, TEL, CLI | 4924 | CC ST21 | V |
| Cj-S-22 | TET | 4924 | CC ST21 | II |
| Cj-M-13* | AZI, CIP, ERY, GEN, FFN, TEL, CLI | 2876 | CC ST21 | II |
| Cj-M-65 | CIP, TET | 2876 | CC ST21 | NM |
| Cj-M-20 | ERY | 4026 | CC ST21 | II |
| Cj-N-33* | AZI, CIP, GEN, TET, NAL, TEL, CLI | 4026 | CC ST21 | II |
| Cj-M-48 | ERY, CLI | 4923 | CC ST21 | V |
| Cj-M-41 | TET, FFN, NAL | 239 | CC ST21 | IV |
| Cj-M-11 | GEN, TET, NAL | 4922 | CC ST21 | II |
| Cj-E-29 | CIP, TET, TEL | 4930 | CC ST21 | II |
| Cj-M-40 | TET | 590 | CC ST42 | IV |
| Cj-M-55 | TET | 590 | CC ST42 | V |
| Cj-E-12 | AZI, TET | 590 | CC ST42 | II |
| Cj-M-60 | TET | 459 | CC ST42 | VII |
| Cj-M-37 | TET | 459 | CC ST42 | III |
| Cj-M-38 | TET, NAL | 459 | CC ST42 | III |
| Cj-M-4 | AZI, TET | 459 | CC ST42 | I |
| Cj-M-2 | TET | 1013 | CC ST42 | I |
| Cj-M-15 | GEN, TET, TEL, CLI | 1013 | CC ST42 | II |
| Cj-M-10 | TET | 1013 | CC ST42 | II |
| Cj-M-46 | TET, NAL | 1013 | CC ST42 | V |
| Cj-M-66 | TET, TEL | 1013 | CC ST42 | NM |
| Cj-M-61 | TET, TEL | 1013 | CC ST42 | VII |
| Cj-M-39 | TET, FFN, CLI | 1013 | CC ST42 | III |
| Cj-N-24 | CIP, GEN, TET, NAL | 1013 | CC ST42 | II |
| Cj-N-47 | AZI, FFN, TEL, CLI | 1013 | CC ST42 | V |
| Cj-S-23 | NAL | 1013 | CC ST42 | II |
| Cj-S-18 | CIP | 1013 | CC ST42 | II |
| Cj-E-58 | AZI, GEN, TET, NAL, TEL | 4929 | Unassigned | VI |
| Cj-E-43 | AZI, ERY, TEL, CLI | 4929 | Unassigned | IV |
| Cj-M-63 | TET | 4925 | Unassigned | VII |
| Cj-M-1 | TET, TEL, CLI | 4931 | Unassigned | I |
| Cj-M-64 | AZI, CIP, ERY, GEN, TET, CLI | 4926 | Unassigned | VII |
| Cj-N-7 | AZI, ERY, FFN, TEL, CLI | 4927 | Unassigned | II |
| Cj-E-57 | CIP, ERY, TET | 922 | Unassigned | VI |
| Cj-E-42 | CIP, GEN, TET, NAL, CLI | 4928 | Unassigned | IV |
| Cj-E-12 | AZI, GEN, TET, CLI | 5448 | Unassigned | II |
| Cj-S-56 | TET | 4932 | Unassigned | V |
| Cj-E-45 | AZI, ERY, GEN, CLI | 61 | CC ST61 | IV |
| Cj-E-59 | CIP, TET, NAL | 61 | CC ST61 | VI |
| Cj-M-17 | AZI, GEN, TET, CLI | 500 | CC ST61 | II |
| Cj-N-26 | CIP, TET, FFN, NAL | 3091 | CC ST45 | II |
| ^a^ Highly susceptible isolates were not presented; these isolates belonged to CC ST21 (n=4), CC ST45 (n=1), CC ST48 (n=1), and unassigned CC (n=2).  ^b^ Macro Restriction profiles. *The antibiotic resistance profiles of two highly invasive *C. jejuni* isolates. | | | | |
